# Bariatric Surgery or Non-surgical Weight Loss for Idiopathic Intracranial Hypertension? A Systematic Review and Comparison of Meta-analyses

**DOI:** 10.1007/s11695-016-2467-7

**Published:** 2016-12-15

**Authors:** James H. Manfield, Kenny K-H. Yu, Evangelos Efthimiou, Ara Darzi, Thanos Athanasiou, Hutan Ashrafian

**Affiliations:** 1 Department of Neurosurgery, Royal Preston Hospital, Preston, Lancashire UK; 2 Department of Bariatric Surgery, Chelsea and Westminster Hospital, London, UK; 3 Department of Surgery and Cancer, Imperial College London, 3rd Floor Chelsea and Westminster Hospital Campus, 369 Fulham Road, London, SW10 9NH UK

**Keywords:** Idiopathic intracranial hypertension, Pseudotumor cerebri, Benign intracranial hypertension, Obesity, Bariatric surgery, Metabolic surgery, Weight loss

## Abstract

**Background:**

Idiopathic intracranial hypertension (IIH) is associated with obesity and weight loss by any means is considered beneficial in this condition.

**Objectives:**

This study aims to appraise bariatric surgery vs. non-surgical weight-loss (medical, behavioural and lifestyle) interventions in IIH management.

**Methods:**

A systematic review and meta-analyses of surgical and non-surgical studies.

**Results:**

Bariatric surgery achieved 100% papilloedema resolution and a reduction in headache symptoms in 90.2%. Non-surgical methods offered improvement in papilloedema in 66.7%, visual field defects in 75.4% and headache symptoms in 23.2%. Surgical BMI decrease was 17.5 vs. 4.2 for non-surgical methods.

**Conclusions:**

Whilst both bariatric surgery and non-surgical weight loss offer significant beneficial effects on IIH symptomatology, future studies should address the lack of prospective and randomised trials to establish the optimal role for these interventions.

**Electronic supplementary material:**

The online version of this article (doi:10.1007/s11695-016-2467-7) contains supplementary material, which is available to authorized users.

## Introduction

The worldwide burden of idiopathic intracranial hypertension (IIH) continues to rise with the current annual incidence estimated at up to 21 per 100,000 per year in obese young women [1]. This increase occurs in the context of a concomitant rise in obesity rates; in the USA, more than a third of adults are now obese, compared with around 11% worldwide, with a further third overweight (body mass index (BMI) 25–30 kg/m^2^). In 2013, the American Medical Association declared obesity as a genuine disease state [2].

IIH, also known as pseudotumour cerebri, is a clinical diagnosis defined by criteria that comprise symptoms and signs of intracranial pressure (e.g. headache, papilloedema and visual loss), elevated intracranial pressure (e.g. on lumbar puncture) with normal cerebrospinal fluid (CSF) composition and without any other cause identified on neuroimaging or other evaluations [3].

Although previously called benign intracranial hypertension, it is not a benign disorder with many patients suffering intractable, disabling headaches with a significant risk of severe and permanent visual loss [4] seen in up to 30% [5].

The pathogenesis of IIH remains unclear, although several risk factors have been identified [6]. IIH is most prevalent in obese females of reproductive age [7]; at least 90% of patients are female with obesity prevalence ranging from 70.5 to 94% [8–10] and recent weight gain is a further significant factor for its development [2].

Weight loss is traditionally advocated for all overweight IIH patients and remains the cornerstone of management as it generally improves symptomology [11]. Although lifestyle weight-loss interventions, comprising exercise promotion and dietary modification are widely advised, long-term weight control and accordingly IIH outcomes remain suboptimal [4].

Bariatric surgery is an alternate way of sustainably reducing both excess weight and IIH symptomology [12], whilst also improving glycaemic control and cardiovascular and cancer risk [13, 14]. A previous review of 65 patients demonstrated that following bariatric surgery 92% (60/65) had improvement in IIH outcomes [15]. Although there is also evidence suggesting that non-surgical interventions, including a recent multicentre RCT of weight loss vs. weight loss with acetazolamide [16], may improve IIH outcomes via weight reduction and possibly additional mechanisms, there are lack of studies directly comparing these treatment strategies.

The aim of this paper was therefore to systematically review the current evidence and concomitantly appraise both bariatric surgery and non-surgical weight-loss interventions in the management of IIH, via the assessment of visual outcomes (papilloedema and visual field deficits), symptoms (headache), intracranial pressure (via cerebrospinal fluid opening pressure measurement) and BMI as summary outcome parameters.

### Methods

The review was performed according to guidelines from the preferred reporting items for systematic reviews and meta-analyses (PRISMA) [17]. A broad search of the electronic literature was performed applying the following search terms: surgical studies: ‘bariatr$ or obesity surg$ or gastr$ surg$’ and ‘intracranial hypertension or pseudotumo$’ and non-surgical studies: ‘weight loss OR weight reduc$’ and ‘intracranial hypertension OR pseudotum$’.

The last date for this search was August 2016. The bibliographies of articles accessed were also reviewed to identify any relevant further literature. Studies included in the final analysis are listed in Table 1 (bariatric surgery) and Table 2 (non-surgical weight-loss management), and this includes non-published data obtained from the corresponding author to facilitate further analysis.

**Table 1:** Surgical weight-loss studies

Study	Study type	Subject number	Average age	Female/male	Procedures performed	Follow-up (months)	Mean BMI (kg/m^2^)		Percentage of subjects with improvement in	
							Pre/post-surgery	Headache	Papilloedema	Visual fields
Sugerman et al. [20]	NRPOS	8	33	8/0	8 RYGB	34	49/27.5	100	100	100
Sugerman et al. [21]	NRPOS	6	32	6/0	5RYGB, 1LGB	<6	45/n/a	83	n/a	n/a
Sugerman et al. [22]	NRPOS	24	34	24/0	23 RYGM, 1 LGB	12	47/30	96	100	n/a
Michaelides et al. [23]	RCS	16	34	16/0	13 RYGB, 3GPs	Various	45/28	81	100^a^	n/a
Nadkarni et al. [24]	RCS	2	42	2/0	1 RYBG, 1 LGB	12	47.9/26.3	100	100	n/a
Egan et al. [25]	NRPOS	4	32	4/0	4 LGB	19.8	46.1/33.4	100	100	50
Sanmugalingam et al. [26]	RCS	5	45	5/0	5 LSG	17	58/37	80	n/a	n/a

Abbreviations: *GP* gastroplasty procedure, *LGB* laparoscopic gastric band, *LSG* laparoscopic sleeve gastrectomy, *NA* not available, *RYGJB* Roux-en-Y gastrojejunostomy bypass, *NRPOS* non-randomised prospective observational study, *RCS* retrospective case series, *RCT* randomised controlled trial

^a^Twelve out of twelve examined

**Table 2:** Non-surgical weight-loss studies

	Study type	Subject number	Average age	Female/male	Follow-up (months)	Pre-interventions	Mean BMI (kg/m^2^)		Percentage of subjects with improvement in		
							Pre/post-interventions	Headache	Papilloedema	Visual fields	Visual symptoms
Wall et al. [16]^a^	RCT	79	30	77/2	6	39.9	39.9/38.6	19.3	38	68	n/a
Newborg [27]	RCS	9	28	7/2	10	42.4	42.4/30.9	n/a	100	n/a	100
Johnson et al. [28]	NRPOS	15	31	15/0	5.5	40.7	40.7/39.2	n/a	73.3	n/a	n/a
Kupersmith et al. [29]	NRPOS	38	n/a	38/0	21.6	n/a	n/a/n/a^e^	n/a	92	89	n/a
Glueck et al. [30]^c^	NRPOS	9	35	9/0	10	37.2	37.2/35.7	87.5	88.9	57	n/a
Ball et al. [31]^a^	RCT	25	33	24/1	12	34.1	34.1/32.9	10	35	n/a	n/a
Sinclair et al. [32]	NRPOS	20	34	20/0	9	38.2	38.2/32.8	45	n/a	n/a	91
Pollak et al. [33]^d^	RCS	82	30	73/9	61.3	31.6	31.6/26	n/a	84^b^	84^b^	n/a

^a^Data from RCT control arm (i.e. weight reduction diet only)

Data also includes personal correspondence from Wall

^b^Composite endpoint (papilloedema and visual fields)

^c^Data from diet only group

^d^Six per cent underwent bariatric surgery; 22% underwent salvage surgery (CSF diversion or optic nerve fenestration)

^e^Data for absolute weight change (kg) available

**Table 3:** Some current hypotheses linking obesity and IIH

Hypothesised factor	Proposed mechanisms	Final common pathway leading to increased CSF pressure and IIH
Increased intra-abdominal pressure (via central obesity).	1. Leads to increased pleural pressure, cardiac filling pressure, and central venous pressure and may lead to increased intracranial venous pressure and IIH [21].	1. Reduced CSF absorption via increased cerebral venous pressure.
	2. Reduced CSF compliance via limited expansion of spinal canal CSF spaces [2].	2. Altered CSF homeostasis.
Hypercoagulable state (obesity is a well-recognised risk factor, which may be at least in party mediated via pro-coagulant adipokines, e.g. leptin [44–46] and sex steroids, e.g. oestrogen [47, 48])	Occult cerebral venous sinus microthrombosis leading to increased cerebral venous pressure and reduced CSF outflow conductance [2, 39].	Reduced CSF absorption
Neuroendocrine adiposopathy (endocrinologically active secretions from adipose tissue include mineralocorticoid releasing factors in addition to the aformentioned adipokines/ sex steroids).	Increased CSF secretion and altered dynamics results from mineralocorticoid receptor activation [5].	Increased CSF secretion

### Inclusion and Exclusion Criteria

All case series and empirical studies that identified patients diagnosed with IIH who underwent either bariatric surgery or conventional weight management approaches were evaluated. Individual case reports were excluded as were manuscripts not reporting outcome data (either symptomatology or visual status) and either BMI or absolute weight change data, as this would preclude further appraisal.

### Data Analysis

The following outcome data was extracted (based on clinical relevance): types of surgery, body weight/BMI and data on symptomatology and signs (visual fields, papilloedema and CSF pressures). Standard deviations, if not explicitly reported, were calculated where possible from available data. Where articles reported multiple follow-up periods, the highest yield interval with the most complete data was selected for inclusion.

Meta-analysis was performed in line with recommendations from the Cochrane Collaboration and followed PRISMA and (MOOSE) guidelines. Continuous data were investigated using weighted mean difference (WMD) reported with 95% confidence intervals (CI). Categorical variables were analysed using risk ratio (RR) with 95% CI. The inverse-variance, random-effect model of DerSimonian and Laird was used for both continuous and categorical outcomes. Interstudy heterogeneity was explored using the *χ* ^2^statistic and the *I* ^2^ statistic. When *I* ^2^ was >65%, significant statistical heterogeneity was considered to be present (*I* ^2^ 30–65% moderate heterogeneity, <30% low heterogeneity). Analysis was performed by use of Stata 13 (StataCorp., College Station, Texas, USA).

**Fig. 1:**
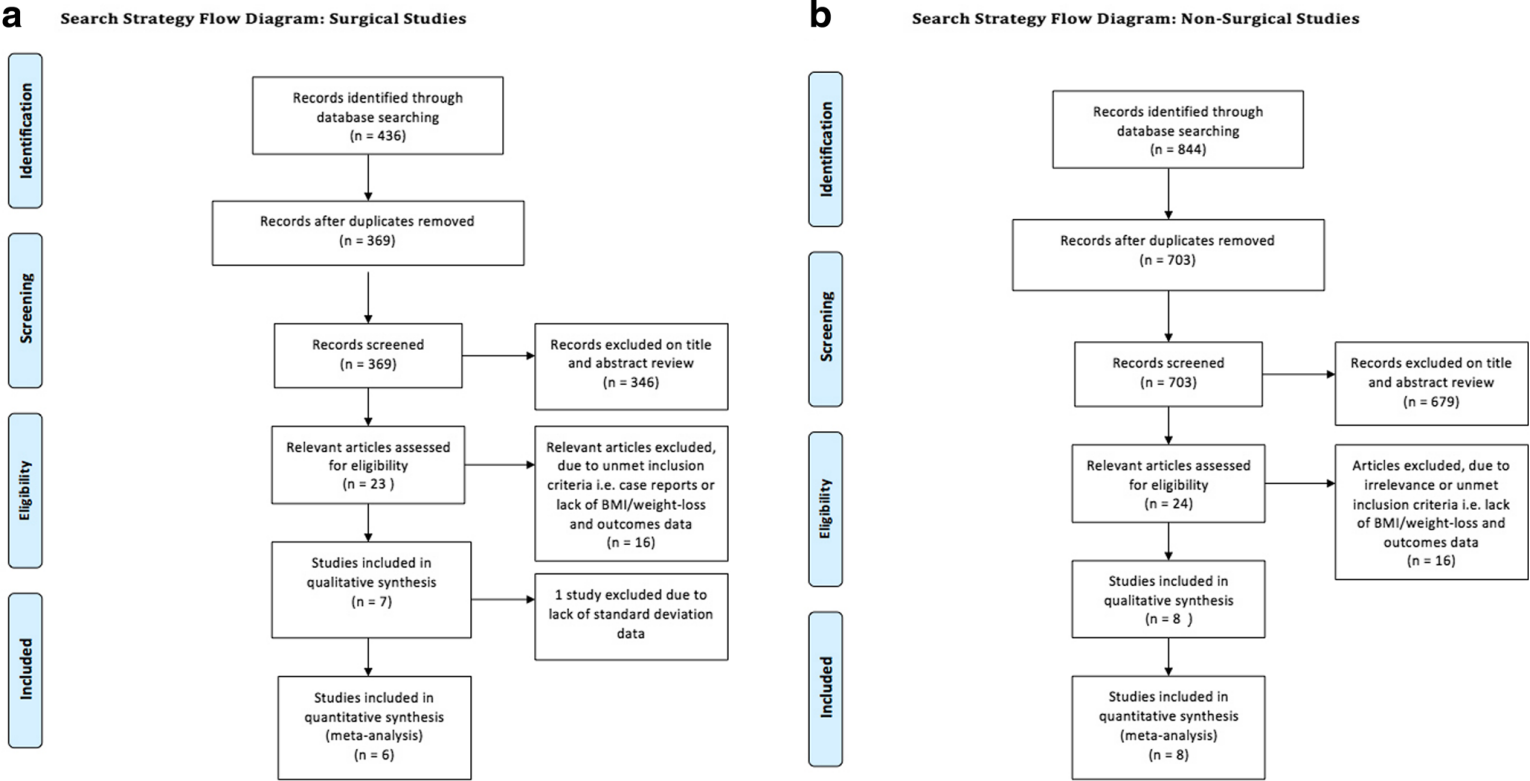
Search strategy flow diagrams for **a** surgical and **b** non-surgical studies

Several strategies were used to evaluate data validity and quality:

Validity was assessed by (1) risk of bias assessments using The Cochrane Collaboration’s tool, (2) funnel plots to assess publication bias and (3) evaluation of publication bias using Egger’s test for small-study effects. Quality scoring was performed using the Newcastle-Ottawa Scale (NOS) [18] for assessing the quality of studies in meta-analyses and the Jadad Scale [19] for randomised trials (see Electronic Supplementary Material for scoring) (Fig. 1).

### Results

#### Surgical Group

Seven studies [20–26] fulfilled the inclusion criteria, generating a pooled data set of 65 patients with IIH undergoing bariatric surgery (see Table 1). Four [20–22, 25] of these were non-randomised prospective observation studies, and three [23, 24, 26] were retrospective case series. One study was subsequently excluded from quantitative synthesis due to lack of standard deviation data precluding further analysis.

#### Non-surgical Group

Eight studies [16, 27–33] met the inclusion criteria, making a pooled data set of 277 patients with IIH undergoing non-surgical management (see Table 2). Two [16, 31] of these were prospective randomised controlled trials, four [28–30, 32] non-randomised prospective observation studies and two [27, 33] retrospective case series.

### Primary Outcomes

#### Papilloedema

##### Surgical Group

Surgical interventions were associated with 100% post-operative resolution.

##### Non-surgical Group

Non-surgical weight-loss intervention was associated with a significant regression in 66.7% (95% CI [45.6, 87.8], *p* = <0.005); interstudy heterogeneity was significant (*χ* ^2^ = 39.4, *p* = 0.000, *I* ^2^ = 84.8%).

#### Visual Field Defects

##### Surgical Group

Only two studies reported these hence data was insufficient for quantitative synthesis. In these two studies, resolution or significant improvement was reported in 100% and 50% of cases, respectively.

##### Non-surgical Group

Non-surgical weight-loss intervention was associated with significant improvement in 75.4% (95% CI, 63.6, 87.2; *p* = <0.005); interstudy heterogeneity was not significant (*χ* ^2^ = 3.67, *p* = 0.300, *I* ^2^ = 18.2%).

#### Headache Symptoms

##### Surgical Group

**Fig. 2:**
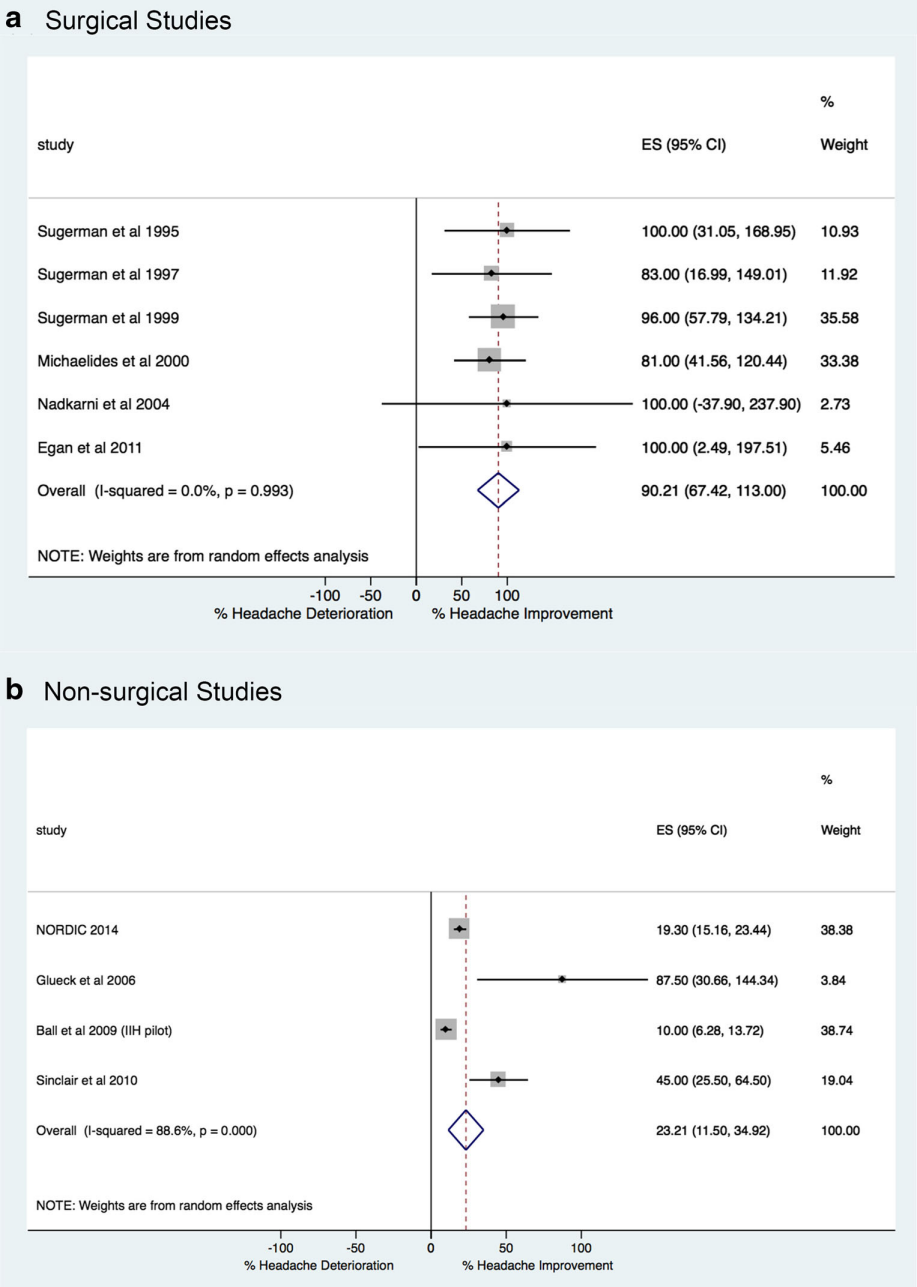
Headache symptom forest plots for **a** surgical and **b** non-surgical studies

Bariatric surgery was associated with a clinically significant post-operative reduction or resolution in 90.2% (95% CI, 67.4, 113; *p* = <0.005) (Fig. 2a); interstudy heterogeneity was not significant (*χ* ^2^ = 0.48, *p* = 0.993, *I* ^2^ = 0.0%).

##### Non-surgical Group

Non-surgical weight-loss intervention was associated with a reduction or resolution in 23.2% (95% CI, 11.5, 34.9; *p* = <0.005) (Fig. 2b); interstudy heterogeneity was significant (*χ* ^2^ = 26.4, *p* = 0.000, *I* ^2^ = 88.6%).

#### Body Mass Index

##### Surgical Group

Surgical intervention was associated with a significant post-operative reduction in BMI of 17.5 kg/m^2^ (95% CI, 14.2, 20.7; *p* = <0.005); interstudy heterogeneity was moderate (*χ* ^2^ = 11.29, *p* = 0.024, *I* ^*2*^ = 64.6%).

##### Non-surgical Group

Non-surgical weight-loss intervention was associated with a significant reduction in BMI of 4.2 kg/m^2^ (95% CI, 1.38, 7.03; *p* = 0.008); interstudy heterogeneity was significant (*χ* ^2^ = 11.8, *p* = 0.019, *I* ^2^ = 66.2%).

#### CSF Pressure

##### Surgical Group

Only two studies reported these hence data were insufficient for quantitative synthesis. In these two studies, CSF pressure decreased in both cases by clinically significant levels (a mean of 185 and 198 mm H_2_0 respectively).

##### Non-surgical Group

Non-surgical weight-loss intervention was associated with a significant reduction in CSF opening pressure of 61.0 mmHg (95% CI, 35.9, 86.0, *p* = <0.005); interstudy heterogeneity was moderate (*χ* ^2^ = 2.12, *p* = 0.145, *I* ^2^ = 52.8%).

### Quality Scoring and Analysis

The overall quality of non-randomised studies is summarised in the Electronic Supplementary Material Tables 1, 2 and 3.

#### Surgical Series

All but one of the seven studies were considered to be of moderate quality scoring ≥ the mean of 6.

#### Non-surgical Series

Of the six studies, three were of moderate quality (scoring 6) and three of high quality (scoring ≥7). There were insufficient high-quality studies to warrant separate subgroup analysis.

The overall results of assessments for each study are also summarised in the Electronic Supplementary Material Tables 3 and 4; most studies were deemed to be at moderate risk of bias with none at critical risk of bias (which would otherwise have warranted exclusion from further analysis).

### Heterogeneity Assessment: Bias Exploration

Funnel plots were employed to detect publication bias (Electronic Supplementary Material Fig. 2). Visual inspection showed no asymmetry, and Egger’s test did not detect a significant small-study effect (Electronic Supplementary Material Fig. 1).

## Discussion

In 65 patients with a mean pre-interventional BMI of 48.3, bariatric surgery gave a weighted mean decrease of BMI by 17.5 kg/m^2^, associated with complete resolution of papilloedema in all documented cases (indicative of relief of raised intracranial pressure) and a significant reduction in headache symptoms. Studies of subjects undergoing non-surgical weight reduction therapies found 277 individuals with a mean pre-intervention BMI of 37.7, which decreased by a weighted mean of 4.2 kg/m^2^. This more modest weight loss was also associated with significant improvements in papilloedema, visual fields and headache symptoms, although excepting visual fields, these were all associated with significant interstudy heterogeneity that was generally not noted in the surgical studies.

This study provides the first available means to systematically and concurrently appraise the effects of surgical and non-surgical weight-loss interventions on BMI and measures of IIH severity. Although surgical and non-surgical patient groups differed in their baseline characteristics, both outcomes of weight loss and the clinical improvement in IIH symptomatology were found to be superior in surgical studies. The quality of non-surgical studies was however higher, comprising class 1 as opposed to class 3 or 4 evidence, such that there is now stronger evidence corroborating the clinical consensus that obesity-associated IIH improves with weight loss. There is also class 1a evidence [34, 35] in the literature that bariatric surgery leads to greater weight loss and higher remission rates of metabolic sequelae compared with non-surgical management, and it is now established as the most effective treatment for morbid obesity and obesity-associated co-morbidities (such as type 2 diabetes mellitus, obstructive sleep apnoea, cardiovascular outcomes, renal dysfunction and cancer), hence more than 340,000 metabolic operations are performed annually worldwide [36–38].

There is now an increasingly accepted view that obesity plays a central role in the development of IIH, although precise pathophysiological mechanisms are not yet fully elucidated [2].

With the largest quantifiable series to date assessing the role of weight loss on IIH, we can confirm that bariatric surgical studies demonstrate a greater effect size on IIH outcomes when relating their results to non-surgical interventions. Although these studies were not comparative trials, and therefore cannot be utilised to convey directly comparative results between bariatric and non-surgical weight-loss interventions, our analysis alludes to the mechanistic effects of sustained weight less in resolving the pathology of IIH.

Current pathogenic theories linking obesity and IIH center on alterations of CSF homeostasis, cerebral venous haemodynamics and other hormonal and metabolic factors [2]. The main hypotheses of IIH aetiology comprise (i) increased cerebral venous pressure, (ii) reduced CSF outflow conductance (both of which result in reduced CSF absorption) and (iii) increased CSF secretion, all of which may be ultimately impacted by obesity [2, 6, 39]. These are summarised in Table 2.

Although metabolic surgery is unlikely to fully replace all measures to manage IIH, as approximately 6–30% of IIH patients are not obese and bariatric operations also pose some operative risk, it offers many advantages compared with other surgical treatment options such as CSF diversion procedures including shunt surgery. These non-bariatric approaches are limited by a high failure which includes symptom recurrence in 48% by 36 months [40] and typical revision rates of at least 30–60% [41–43]. Bariatric surgery has the advantage of directly targeting obesity and its associated metabolic dysfunction as well as mitigating other obesity-related co-morbidities.

Further work is however necessary to clarify whether bariatric surgery offers an equivalence in the rapidity of treatment outcome in the context of acute or rapidly progressive visual loss, given the time required for both multidisciplinary pre-operative workup and resolution of symptoms [12].

We suggest that IIH can be regarded both as significant, and as a condition convincingly demonstrated to respond to weight loss, and as such it is reasonable for bariatric surgery to be offered to patients in this group. As such we explicitly advocate that IIH should be considered as a co-morbidity of obesity that should be added to the criteria for bariatric surgery worldwide (it is principally listed only in clinical practice guidelines in the USA for patients with IIH and BMI > =35 kg/m^2^ [15]).

It is also noteworthy that multiple studies have associated more severe obesity with worse visual outcomes in IIH [5] which further supports the rationale for aggressive treatment in morbidly obese individuals. Recently, an RCT has been commenced comparing bariatric surgery vs. a community weight-loss programme for the sustained treatment of IIH (NCT02124486). This will likely clarify some factors regarding patient selection for bariatric surgery in IIH; however, remaining questions in this field include: (a) Whether bariatric procedures should be a first line option for a selected obese patients, (b) which bariatric procedure is most preferable in these patients and (c) the most appropriate BMI cutoff where the benefits of surgery outweigh possible risks (0.08% mortality within 30 days and a reoperation rate of 7% [13]).

## Strengths and Limitations

This meta-analysis statistically appraises pooled data from 65 patients in 7 surgical studies and 277 patients receiving non-surgical management in 8 studies, which is the largest synthesis to date. There are nevertheless several limitations within which context these results should be interpreted. Most of the constituent studies are intrinsically limited by their design with no surgical studies and two non-surgical studies being randomised controlled trials. Surgical studies are further limited to uncontrolled series although we have omitted the multiple case reports as these are inherently biased towards favourable outcomes. Nonetheless, the effect size of surgical intervention was marked and without significant heterogeneity, in contrast to that seen in non-surgical interventions which are indicative of several potential confounding variables. Despite this heterogeneity in the non-surgical group, we performed an analysis based on the aggregation of interventions as this reflects clinic practice where lifestyle approaches (e.g. diet and exercise programs) are typically practised concurrently. Furthermore, other studies have utilised this approach as an established methodology [37].

Our study aimed to clarify the impact of weight loss on IIH outcomes, rather than the method by which the weight loss is achieved; hence, we have included studies where this is quantified. Of the seven non-surgical studies included the primary intervention was a specified low-energy diet in four [16, 27, 30, 32] as opposed to weight loss via unspecified means in the other three. In the three [16, 30, 31] studies with multiple arms, data from the weight-loss-only arm was used for analysis. Overall, co-interventions were adequately appraised with clinical or statistical controls.

Studies in both arms differed in demographics, follow-up period and spanned a 40-year time period. This could mean our analysis may not capture the difference between contemporary and historic weight-loss modalities or the developments in IIH diagnostic workup (particularly higher resolution neuroimaging which can exclude differential diagnoses). For instance, bariatric surgical techniques have evolved, yet the newer technique of sleeve gastrectomy is under-represented in these studies and some of our analysed data comes from gastroplasty, which is no longer routinely performed. Similar inconsistencies are present in non-surgical studies, and both arms also included patients that had undergone cerebrospinal fluid diversion therapies or optic nerve sheath fenestration (another salvage procedure for deteriorating visual function).

Patients in both the surgical and non-surgical trials (excluding the two non-surgical RCTs who used a placebo) received the diuretic acetazolamide which has been shown to have a modest effect on IIH symptoms [16]. Other non-surgical weight-loss interventions were non-standardised between trials, reflecting real world variation in practice. A small number of non-surgical patients also underwent bariatric surgery in one included study.

Studies in both arms also showed variation in reported outcome measures, which restricted the extent of analysis; notably in the case of visual field status and CSF opening pressure following bariatric surgery. In comparing the two meta-analytical groups, both the mean pre-intervention BMI and the prevalence of IIH symptoms and signs were materially higher in the surgical group, which could impact the overall reduction in BMI and degree of decrease in symptoms (although absolute improvements were still considerably greater than in non-surgical patients).

Though this analysis elucidates the comparison of surgical and non-surgical studies by combining results from both treatment strategies, it does not formally quantify the difference in their effects. As result of the selection criteria requiring the inclusion of both BMI and visual status findings pre and post-intervention, several studies were excluded meaning that our analysis may not be fully representative of all interventions in the literature to date.

## Conclusion

We demonstrate that both bariatric surgery and non-surgical weight loss may benefit IIH patients to improve papilloedema and headache symptoms. Bariatric surgery offers a materially greater treatment effect, in addition to the health benefits of significant sustained weight loss and metabolic improvement.

The current evidence base is limited by a lack of randomised controlled surgical trials and comparative studies between surgical and non-surgical treatment strategies. Nevertheless, there is broad consensus that obesity plays a central role in the pathogenesis of IIH and weight loss remains the essential cornerstone of management. Bariatric surgery is the most effective method achieving sustainable weight loss in obese patients. Other treatment strategies, such as CSF diversion and optic nerve sheath fenestration, are limited by a high incidence of complications and they do not treat the most significant and modifiable underlying risk factor, i.e. obesity.

Based on the best evidence available, a compelling case can be made to regard IIH as obesity co-morbidity and thus bariatric surgery should be offered at similar BMI thresholds to other obesity co-morbidities in line with internationally endorsed guidelines.

Further research is needed to determine the precise BMI threshold where the benefits of bariatric surgery outweigh its short- and long-term operative risks, as well as cost effectiveness. This requires a holistic consideration of all the consequences of obesity of the patient rather than only IIH in isolation. More and better-designed trials are now required to evaluate post-intervention periods, effects on visual loss and underlying mechanistic factors to establish the precise relationship between bariatric surgery and non-surgical weight-loss management in IIH resolution.

## Electronic Supplementary Material

ESM 1 (DOCX 15 kb.)

ESM 2 (DOCX 13 kb)

ESM 3 (DOCX 13 kb)

ESM 4 (DOCX 13 kb)

ESM 5 (DOCX 59 kb)

ESM 6 (DOCX 173 kb)
