# Supplementary material for: Bariatric Surgery or Non-surgical Weight Loss for Idiopathic Intracranial Hypertension? A Systematic Review and Comparison of Meta-analyses
Source: Obes Surg. 2016 Dec 15;27(2):513–21. doi: 10.1007/s11695-016-2467-7 (PMC5237659; doi:10.1007/s11695-016-2467-7)
Supplement: Supplementary file 1 — (DOCX 15 kb.) [file 11695_2016_2467_MOESM1_ESM.docx]

**Supplementary Appendix**

**Table 1:** Newcastle-Ottowa Quality Scoring (Non Randomised Studies)

| **Author** | | **Selection** | | **Compar-**  **ability** | | | **Outcome** | |  | **Total** | | |
| --- | --- | --- | --- | --- | --- | --- | --- | --- | --- | --- | --- | --- |
|  |  | **1** | **2** | **3** | **4** | **5** | 6 | **7** | | **8** |  |  |
|  | **(a) Bariatric Surgery** | | | | | | | | | | | |
| Sugerman et al 1995 | | * | - | * | * | - | * | * | | * | 6 |  |
| Sugerman et al 1997 | | * | - | * | - | - | * | - | | * | 4 |  |
| Sugerman et al 1999 | | * | - | * | * | - | * | * | | * | 6 |  |
| Michaelides et al 2000 | | * | - | * | * | - | * | * | | * | 6 |  |
| Nadkarni et al 2004 | | * | - | * | * | - | * | * | | * | 6 |  |
| Egan et al 2011 | | * | - | * | * | - | * | * | | * | 6 |  |
| Sanmugalingam et al 2013 | | * | - | * | * | - | * | * | | * | 6 |  |
|  | **(b) Non-Surgical Weight Loss Intervention** | | | | | | | | | | | |
| Newborg 1974 | | * | - | * | * | - | * | * | | * | 6 |  |
| Johnson et al 1998 | | * | * | * | * | - | * | * | | * | 7 |  |
| Kupersmith et al 1998 | | * | * | * | * | * | * | * | | * | 8 |  |
| Glueck et al 2006 | | * | * | * | * | * | * | * | | * | 8 |  |
| Sinclair et al 2010 | | * | - | * | * | - | * | * | | * | 6 |  |
| Pollak et al 2013 | | * | - | * | * | - | * | * | | * | 7 |  |
