# Supplementary material for: Bariatric Surgery or Non-surgical Weight Loss for Idiopathic Intracranial Hypertension? A Systematic Review and Comparison of Meta-analyses
Source: Obes Surg. 2016 Dec 15;27(2):513–21. doi: 10.1007/s11695-016-2467-7 (PMC5237659; doi:10.1007/s11695-016-2467-7)
Supplement: Supplementary file 2 — (DOCX 13 kb) [file 11695_2016_2467_MOESM2_ESM.docx]

**Supplementary Appendix**

**Table 2:** Jadad Quality Scoring (Randomised Studies)

| **Author** | | **Randomisation** | | | **Blinding** | | | **Account of all patients** | | |  | | **Total** | |
| --- | --- | --- | --- | --- | --- | --- | --- | --- | --- | --- | --- | --- | --- | --- |
|  |  |  |  | |  |  |  | |  |  | |  | |  |
|  |  | | | | | | | | | | | | | |
| Ball et al 2009 | | 2 | | - | | | 1 | | | | | 3 | | |
| Wall et al 2014 | | 2 | | 2 | | | 1 | | | | | 5 | | |
