# Supplementary material for: Bariatric Surgery or Non-surgical Weight Loss for Idiopathic Intracranial Hypertension? A Systematic Review and Comparison of Meta-analyses
Source: Obes Surg. 2016 Dec 15;27(2):513–21. doi: 10.1007/s11695-016-2467-7 (PMC5237659; doi:10.1007/s11695-016-2467-7)
Supplement: Supplementary file 3 — (DOCX 13 kb) [file 11695_2016_2467_MOESM3_ESM.docx]

**Supplementary Appendix**

**Table 3:** Overall Risk of Bias assessments for non-randomised studies of interventions (Cochrane ACROBAT-NRSI)

| **Author** | **Overall Risk of Bias** |  |  |  |  |
| --- | --- | --- | --- | --- | --- |
| **(a) Bariatric Surgery** | | | | | |
| Sugerman et al 1995 | Moderate | | | | |
| Sugerman et al 1997 | Serious | | | | |
| Sugerman et al 1999 | Moderate | | | | |
| Michaelides et al 2000 | Moderate | | | | |
| Nadkarni et al 2004 | Moderate | | | | |
| Egan et al 2011 | Serious | | | | |
| Sanmugalingam et al 2013 | Insufficient data to assess | | | | |
| **(b) Non-Surgical Weight Loss Intervention** | | | | | |
| Newborg 1974 | Moderate | | | | |
| Johnson et al 1998 | Moderate | | | | |
| Kupersmith et al 1998 | Moderate | | | | |
| Glueck et al 2006 | Moderate | | | | |
| Sinclair et al 2010 | Moderate | | | | |
| Pollak et al 2013 | Serious | | | | |
