# Supplementary material for: Bariatric Surgery or Non-surgical Weight Loss for Idiopathic Intracranial Hypertension? A Systematic Review and Comparison of Meta-analyses
Source: Obes Surg. 2016 Dec 15;27(2):513–21. doi: 10.1007/s11695-016-2467-7 (PMC5237659; doi:10.1007/s11695-016-2467-7)
Supplement: Supplementary file 4 — (DOCX 13 kb) [file 11695_2016_2467_MOESM4_ESM.docx]

**Supplementary Appendix**

**Table 4:** Overall Risk of Bias assessments for randomised trials (The Cochrane Colaboration’s tool for assessing risk of bias in randomized trials).

| **Author** | **Overall Risk of Bias** |  |  |  |  |
| --- | --- | --- | --- | --- | --- |
| **Non-Surgical Weight Loss Intervention** | | | | | |
| Ball et al 2011 | Unclear | | | | |
| Wall et al 2014 | Low | | | | |
