# Supplementary material for: Bariatric Surgery or Non-surgical Weight Loss for Idiopathic Intracranial Hypertension? A Systematic Review and Comparison of Meta-analyses
Source: Obes Surg. 2016 Dec 15;27(2):513–21. doi: 10.1007/s11695-016-2467-7 (PMC5237659; doi:10.1007/s11695-016-2467-7)
Supplement: Supplementary file 5 — (DOCX 59 kb) [file 11695_2016_2467_MOESM5_ESM.docx]

**Supplementary Appendix**

**Figure 1: Egger’s Tests for small-study effects:**

**Surgical Studies**

**
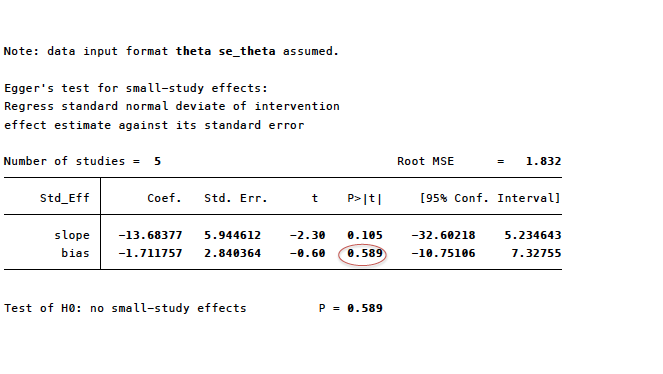
**

**Non-surgical studies**

**
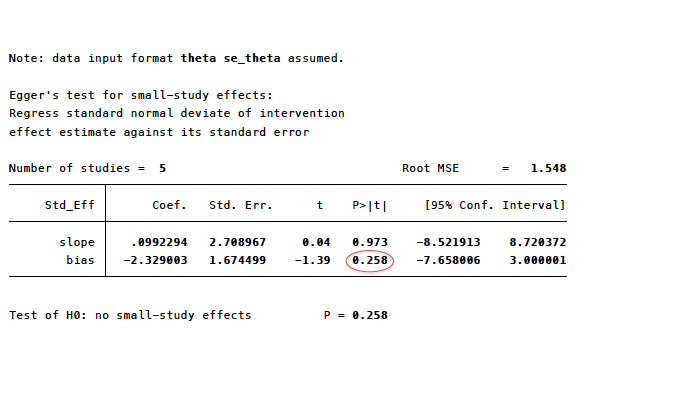
**
